# Pharmacological and therapeutic potential of *Cordyceps* with special reference to Cordycepin

**DOI:** 10.1007/s13205-013-0121-9

**Published:** 2013-02-19

**Authors:** Hardeep S. Tuli, Sardul S. Sandhu, A. K. Sharma

**Affiliations:** 1 Department of Biotechnology, M.M.E.C., Maharishi Markandeshwar University, Mullana, Ambala, 133203 Haryana India; 2 Department of Biological Sciences, R. D. University, Jabalpur, 482001 MP India

**Keywords:** *Cordyceps militaris*, Infection, Cordycepin, Mechanism, Pharmacological effect

## Abstract

An entomopathogenic fungus, *Cordyceps* sp. has been known to have numerous pharmacological and therapeutic implications, especially, in terms of human health making it a suitable candidate for ethno-pharmacological use. Main constituent of the extract derived from this fungus comprises a novel bio-metabolite called as Cordycepin (3′deoxyadenosine) which has a very potent anti-cancer, anti-oxidant and anti-inflammatory activities. The current review discusses about the broad spectrum potential of Cordycepin including biological and pharmacological actions in immunological, hepatic, renal, cardiovascular systems as well as an anti-cancer agent. The article also reviews the current efforts to delineate the mechanism of action of Cordycepin in various bio-molecular processes. The study will certainly draw the attention of scientific community to improve the bioactivity and production of Cordycepin for its commercial use in pharmacological and medical fields.

## Introduction

Medicinal mushrooms have been known for thousands of years to produce biometabolites which are used or studied as possible treatment for diseases. Over two-third of cancer-related deaths could be prevented or reduced by modifying our diet with mushrooms, as they contain anti-oxidants (Borchers et al. 2004; Zaidman et al. 2005). *Cordyceps* have a history of medicinal use spanning millennia in parts of Asia (Gu et al. 2007). The name *Cordyceps* has been derived from two Latin words, i.e., *cord* and *ceps* meaning club and head, respectively. *Cordyceps militaris* belongs to the phylum Ascomycota classified in the order hypocreales, as spores are produced internally inside a sac, called ascus (Wang et al. 2008). It is an entomopathogenic fungus having an annual appearance which often grows parasitically on lepidopteron larvae and pupae of insects and spiders. It normally inhabits on the surface of insects pupae in winters and leading to the formation of fruiting body in summers justifying its name as “winter-worm summer-grass”.

*Cordyceps* has been found mainly in North America, Europe and Asia (Mains 1958; Winkler 2010; Panda and Swain 2011). In India, it is prominently found in subalpine regions of grassy lands of Himalayas commonly known as “Keera Ghas”. Recently it has been reported from Sutol and Kanol villages of Chamoli district of Uttarakhand (Singh et al. 2010). The ethnopharmacological use of *Cordyceps sinensis* has been reported from western Nepal for the cure of different diseases like diarrhea, headache, cough, rheumatism, liver disease, etc. This herb is also referred as “Himalayan Viagra” or “Himalayan Gold” due to its broad clinical and commercial value (Devkota 2006). *Cordyceps* requires specific set of conditions for its growth and has small size; therefore, the large-scale collection of this mushroom is a daunting task. However, people within the age group 15–65 years including men, women, young boys and girls are the main collectors of this fungus and price for 1 kg of wild-collected mushroom in the market of Nepal varies from 30,000 to 60,000 Nepali Rupees while in India it costs about Rupees 100,000 (Sharma 2004). Past 5 years have seen tremendous exploitation of *Cordyceps* which has significantly reduced its wild occurrence (Negi et al. 2006; Winkler 2008). Efforts have been made to artificially cultivate this mushroom by surface and submerged fermentation techniques.

**Fig. 1:**
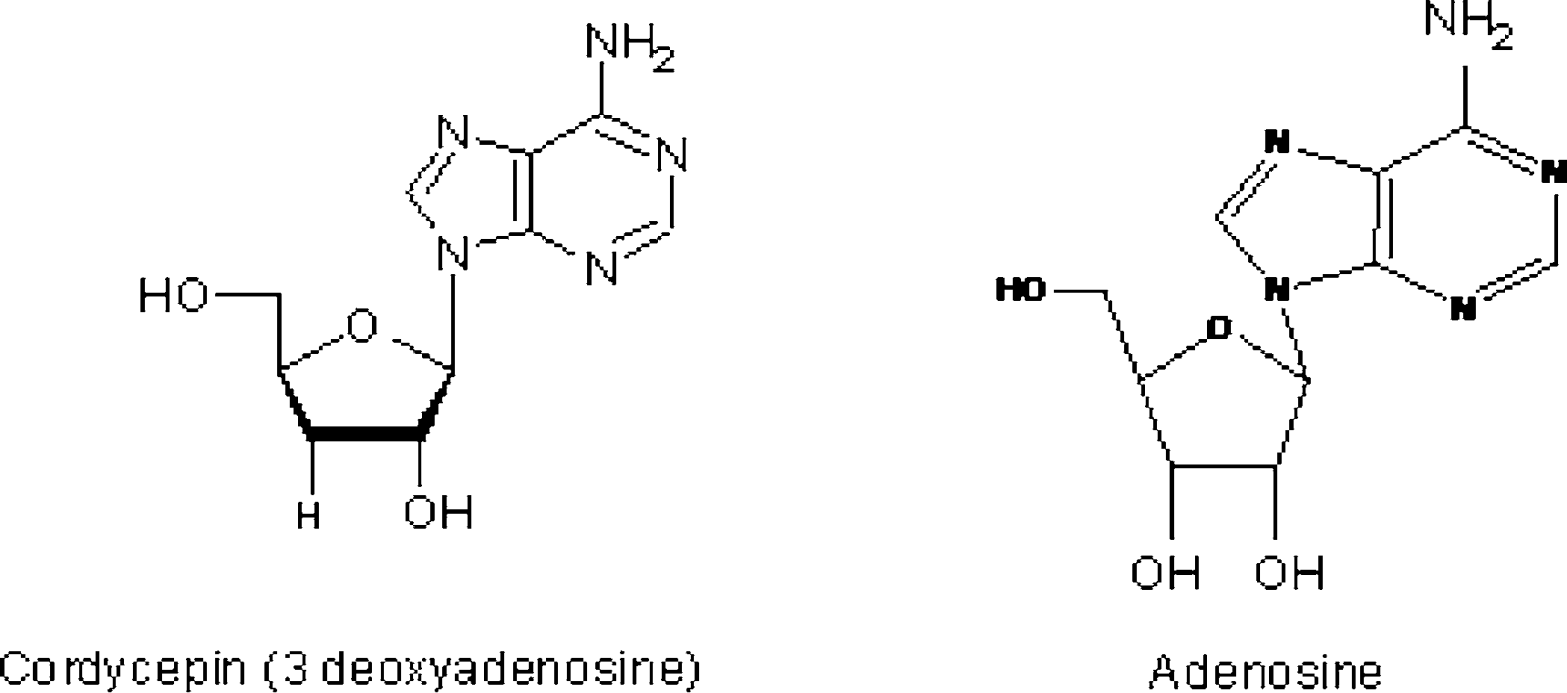
The figure elucidates the difference in the chemical structures of bioactive compounds, Cordycepin and adenosine, produced by *Cordyceps militaris*

**Fig. 2:**
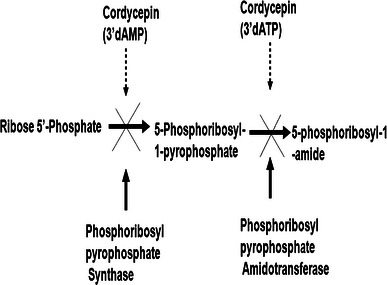
The inhibitory effect of Cordycepin in mono- and tri- phosphate states on the enzymes, phosphoribosyl pyrophosphate synthase and phosphoribosyl pyrophosphate amidotransferase, involved in purine biosynthesis pathway

**Fig. 3:**
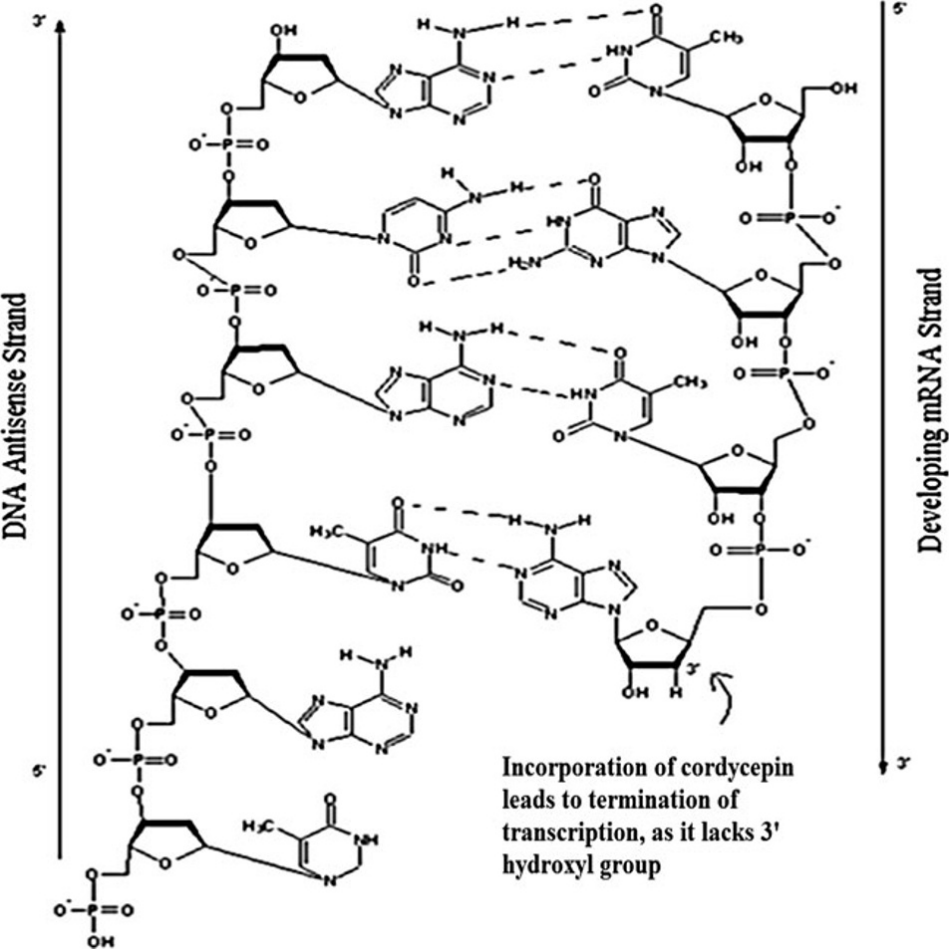
The addition of Cordycepin as a Co-TP (Cordycepin tri-phosphate) leads to transcriptional termination

**Fig. 4:**
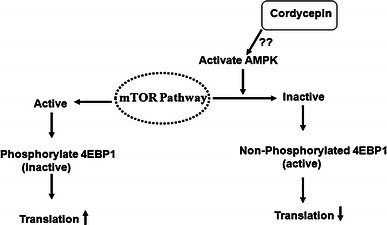
Cordycepin presumably activates the AMPK by some unknown mechanism which further negatively regulates the translation of mTOR signaling transduction pathway by the formation of a translational repressor, 4-E-binding protein-1 (4EBP1)

There have been a variety of pharmacologically active compounds (e.g., Cordycepin) reported from *Cordyceps* sp. Cordycepin (Fig. 1) has received much attention due to its broad-spectrum biological activity. It is known to interfere with various biochemical and molecular processes including purine biosynthesis (Fig. 2) (Overgaard 1964; Rottman and Guarino 1964), DNA/RNA synthesis (Fig. 3) (Holbein et al. 2009) and mTOR (mammalian target of rapamycin) signaling transduction (Fig. 4) (Wong et al. 2010). *Cordyceps* has been included as one of the growing numbers of fungal traditional Chinese medicine (FTCM) used as cures for modern diseases with many products available commercially. Due to recent advancements in pharmaceutical biotechniques, it is possible to isolate bioactive compounds from *Cordyceps* and make it available in powder as well as in capsular form (e.g., Didanosine). *Cordyceps* and its product have remarkable clinical health effects including action on hepatic, renal, cardiovascular, respiratory, nervous, sexual, immunological systems, besides having anti-cancer, anti-oxidant, anti-inflammatory and anti-microbial activities (Zhou et al. 2008; Wang et al. 2011; Lee et al. 2011a, b; Zhang et al. 2012; Patel and Goyal 2012; Yue et al. 2012).

Keeping in view of the above facts, the current review updates us with the recent research pertaining to *Cordyceps* and the bioactive compounds isolated from it; especially for its ethno-pharmacological use. The study brings together a variety of mechanisms of Cordycepin at one platform and more importantly the broad spectrum pharmacological, clinical or biological activities associated with *Cordyceps*.

## Infection to the host

*Cordyceps* usually infects insects at different stages of their development ranging from insect larvae to adult. Insect’s epidermis is covered with a thick layer of cuticle (procuticle and epicuticle) which is also known as integument. Insect’s integument comprises chitin, proteins and lipids. Beside this, it also contains variety of enzymes and phenolic compounds (Leger et al. 1991). Epidermis is formed by a single layer of epithelial cells followed by a thick layer of procuticle. Procuticle is differentiated into an inner soft part known as an endocuticle while the outer hard part is called exocuticle. Epicuticle and wax are known to constitute the outermost covering of the cuticle. This not only serves as a protective barrier against pathogenic organisms but also prevents water loss and acting as an interface between insect and its environment. Out of all these components, chitin which is a kind of heteropolysaccharide made with the polymerization of *N*-acetyl glucosamine through 1–4 β-linkage constitutes an important structural component of insect’s integument. Pathogen has to invade this tough integument covering to gain entry into the host.

Infection begins with the dispersion of fungus conidia on insect’s surface. Once conidia get settled, they start germinating within a few hours under suitable conditions. To get protection from the environmental ultraviolet radiations, protective enzymes like Cu–Zn superoxide dismutase (SOD) and peroxidases are secreted by the fungal conidia. These enzymes provide protection to the conidia from reactive oxygen species (ROS) generated due to UV rays and heat in the environment (Wanga et al. 2005). Besides this, conidia secrete certain hydrolytic enzymes like proteases, chitinases and lipases which lead to the dissolution of the integument and play a very important role in infection to the host. These enzymes not only provide a penetration path to the conidia but also provide nutrition to the germinating conidia (Ali et al. 2010).

Further a short germ tube protruding out of the conidia starts thickening at the distal end which is known as appressorium. This appressorium maintains a kind of mechanical pressure on the germinating germ tube further improving the penetration effect of germ tube so as to reach into the insect’s haemolymph (Hajek and Leger 1994). As the germ tube penetrates the epicuticle layer of insect’s integument, it starts forming a plate-like structure called penetration plate. The penetration plate further produces secondary hyphae, which cross the epidermal layer and reach into the haemocoel of insect’s body. From these hyphae, protoplast bodies bud off and start circulating into the insect’s haemocoel. Fungus now starts growing into a filamentous mode invading internal organs and tissues of the host. During growth inside the host, fungus produces various kinds of toxic secondary metabolites, which are insecticidal. These secondary metabolites take the insect to its final life stage and ultimately insect dies out. Fungal mycelium emerges out through the cuticle and lead to the formation of fruiting body under suitable environmental conditions (Webster 1980). Morphological features of fruiting body include stipitate, yellowish-orange to orange to reddish-orange fruiting stroma which is cylindrical to slightly clavate in shape. Stipes of 1.5- to 3-mm thickness with fertile clava terminal (2.0- to 6.0-mm wide) are also commonly seen in the fruiting body with overall stroma of about 1.5- to 7.0-cm tall which can vary in length depending on the size of the host.

### *Cordyceps* diversity and cultivation

There are more than 1,200 entomopathogenic fungi reported (Humber 2000) in the literature out of which the *Cordyceps* constitutes one of the largest genus containing approximately 500 species and varieties (Hodge et al. 1998; Hywel 2002; Muslim and Rahman 2010). Many different species of *Cordyceps* are being cultivated for their medicinal and pharmaceutical properties including *O. sinensis, C. militaris, C. ophioglossoides, C. sobolifera, C. liangshanesis,* and *C. cicadicola*. Similarly many other species of *Cordyceps* have been documented like *C. tuberculata*, *C. subsessilis, C. minuta, C. myrmecophila, C. Canadensis*, *C. agriota, C. gracilis, C. ishikariensis, C. konnoana, C. nigrella, C. nutans, C. pruinosa, C. scarabaeicola, C. sphecocephala, C. tricentri*, etc., although the molecular evidence for their proper phylogenetic placement is still lacking (Shrestha and Sung 2005; Wang et al. 2008; Zhou et al. 2009).

Nearly 80–85 % of all medicinal mushroom products are extracted from their fruiting bodies while only 15 % are derived from mycelium culture (Lindequist et al. 2005). Fruiting body of *Cordyceps* is a very small blade-like structure, making its collection difficult and expensive. Since there is a huge requirement of medicinal mushroom bio-metabolites, it is necessary to cultivate mycelium biomass artificially for which variety of methods for its cultivation have been proposed by many research groups (Masuda et al. 2006; Das et al. 2008, 2010a). *Cordyceps* mycelium can grow on different nutrients containing media, but for commercial fermentation and cultivation, insect larvae (silkworm residue) and various cereal grains have been used in the past. It has been seen consistently that from both insect larvae and cereal grains, fruiting body of fungus can be obtained with almost comparable medicinal properties (Holliday et al. 2004).

There are basically two fermentation techniques by which the cultivation of mycelium biomass of *Cordyceps* can be achieved including surface and submerged fermentation. In surface fermentation, the cultivation of microbial biomass occurs on the surface of liquid or solid substrate. This technique, however, is very cumbersome, expensive, labor intensive and rarely used at the industrial scale. While in submerged fermentation, micro-organisms are cultivated in liquid medium aerobically with proper agitation to get homogenous growth of cells and media components. However, there is a loss of extra-cellular compounds (after harvesting mycelium) from the broth which makes it necessary to improve the culture medium composition and downstream processing technology to get large-scale production of the secondary bio-metabolites (Ni et al. 2009). It has been observed that the highest productivity can be achieved by repeated batch culture technique in which waste medium is removed at the end of the process and further refreshing the medium gives higher productivity of cells and bio metabolites.

### Nutritional value of *Cordyceps*

In *Cordyceps*, there occurs a wide range of nutritionally important components including various types of essential amino acids, vitamins like B1, B2, B12 and K, different kinds of carbohydrates such as monosaccharide, oligosaccharides and various medicinally important polysaccharides, proteins, sterols, nucleosides, and other trace elements (Hyun 2008; Yang et al. 2009, 2010; Li et al. 2011). In the fruiting body and in the corpus of *C. militaris*, the reported total free amino acid content is 69.32 and 14.03 mg/g, respectively. The fruiting body harbors many abundant amino acids such as lysine, glutamic acid, proline and threonine as well. The fruiting body is also rich in unsaturated fatty acids (e.g., linoleic acid), which comprises of about 70 % of the total fatty acids. There are differences in adenosine (0.18 and 0.06 %) and Cordycepin (0.97 and 0.36 %) contents between the fruiting body and the corpus, respectively (Hyun 2008).

### Bio-metabolites isolated from *Cordyceps*

*Cordyceps*, especially its extract has been known to contain many biologically active compounds like Cordycepin, cordycepic acid, adenosine, exo-polysaccharides, vitamins, enzymes etc. (Table 1). Out of these, Cordycepin, i.e., 3′-deoxyadenosine (Fig. 1) isolated from ascomycetes fungus *C. militaris*, is the main active constituent which is most widely studied for its medicinal value having a broad spectrum biological activity (Cunningham et al. 1950).

**Table 1:** Bioactive compounds isolated from *Cordyceps* sp.

S. no	Bioactive compounds	References
1	Cordycepin	Cunningham et al. (1950)
2	Cordycepic acid	Chatterjee et al. (1957)
3	*N*-acetylgalactosamine	Kawaguchi et al. (1986)
4	Adenosine	Guo et al. (1998)
5	Ergosterol and ergosteryl esters	Yuan et al. (2007)
6	Bioxanthracenes	Isaka et al. (2001)
7	Hypoxanthine	Huang et al. (2003)
8	Acid deoxyribonuclease	Ye et al. (2004)
9	Polysaccharide and exopolysaccharide	Yu et al. (2007, 2009), Xiao et al. (2010), Yan et al. (2010)
10	Chitinase	Lee and Min (2003)
11	Macrolides (C_10_H_14_O_4_)	Rukachaisirikul et al. (2004)
12	Cicadapeptins and myriocin	Krasnoff et al. (2005)
13	Superoxide dismutase	Wanga et al. (2005)
14	Protease	Hattori et al. (2005)
15	Naphthaquinone	Unagul et al. (2005)
16	Cordyheptapeptide	Rukachaisirikul et al. (2006)
17	Dipicolinic acid	Watanabe et al. (2006)
18	Fibrynolytical enzyme	Kim et al. (2006)
19	Lectin	Jung et al. (2007)
20	Cordymin	Wonga et al. (2011)

### Cordycepin: mechanism of action

The structure of Cordycepin is very much similar with cellular nucleoside, adenosine (Fig. 1) and acts like a nucleoside analogue.

### Inhibition of purine biosynthesis pathway

Once inside the cell, Cordycepin gets converted into 5′ mono-, di- and tri-phosphates that inhibit the activity of enzymes like ribose-phosphate pyrophosphokinase and 5-phosphoribosyl-1-pyrophosphate amidotransferase which are used in de novo biosynthesis of purines (Fig. 2) (Klenow 1963; Overgaard 1964; Rottman and Guarino 1964).

### Cordycepin provokes RNA chain termination

Cordycepin lacks 3′ hydroxyl group in its structure (Fig. 1), which is the only difference from adenosine. Adenosine is a nitrogenous base and acts as cellular nucleoside, which is needed for the various molecular processes in cells like synthesis of DNA and/or RNA. During the process of transcription (RNA synthesis), some enzymes are not able to distinguish between an adenosine and Cordycepin which leads to incorporation of 3′-deoxyadenosine or Cordycepin, in place of normal nucleoside preventing further incorporation of nitrogenous bases (A, U, G, and C), leading to premature termination of transcription (Fig. 3) (Chen et al. 2008; Holbein et al. 2009).

### Cordycepin interferes in mTOR signal transduction

Cordycepin has been reported to shorten the poly A tail of m-RNA which further affects its stability inside the cytoplasm. It was observed that inhibition of polyadenylation with Cordycepin of some m-RNAs made them more sensitive than the other mRNAs. At higher doses, Cordycepin inhibits cell attachment and reduces focal adhesion. Further increase in the dosage of Cordycepin may shutdown mTOR (mammalian target of rapamycin) signaling pathway (Fig. 4) (Wong et al. 2010). The name mTOR has been derived from the drug rapamycin, because this drug inhibits mTOR activity. The mTOR inhibitors such as rapamycin and CCI-779 have been tested as anti-cancer drugs, because they inhibit or block mTOR signaling pathway. mTOR is a 298 kDa serine/threonine protein kinase from the family PIKK (Phosphatidylinositol 3-kinase-related kinase). The mTOR plays a very important role to regulate proteins synthesis. However, mTOR itself is regulated by various kinds of cellular signals like growth factors, hormones, nutritional environment, and cellular energy level of cells. As growth factors bind with cell receptor, Phosphatidyl inositol 3 kinase (PI3K) gets activated, converts phosphatidyl inositol bisphosphate (PIP2) to phosphatidyl inositol trisphosphate (PIP3). PIP3 further activates PDK1 (phosphoinositide dependent protein kinase 1). The activated PDK1 then phosphorylates AKT 1 kinase and makes it partially activated which is further made fully activated by mTORC2 complex. The activated AKT 1 kinase now activates mTORC1 complex that leads to the phosphorylation of 4EBP1 (translational repressor) and makes it inactive, switching on the protein synthesis (Wong et al. 2010). The study confirmed that under low nutritional stress, Cordycepin activates AMPK which blocks the activity of mTORC1 and mTORC2 complex by some unknown mechanism. The inactivated mTORC2 complex cannot activate AKT 1 kinase fully, which in turn blocks mTOR signal transduction inhibiting translation and further cell proliferation and growth (Fig. 4).

## Molecular studies of genes isolated from *Cordyceps* sp.

It is necessary to understand the genetic makeup and molecular biology of *Cordyceps* not only to enhance the production of Cordycepin and exopolysaccharides but also to figure out the biochemical synthetic pathway of the above bio-metabolites. Cordycepin and exopolysaccharides are some of the major pharmacologically active constituents of *Cordyceps*. There exists a variety of valuable genes encoding enzymes isolated and subsequently cloned from this medicinally important insect fungus. Isolation and cloning of *FKS1* gene has been carried out successfully from *Cordyceps* which encodes for an integral membrane protein acting as a catalytic subunit for enzyme β-1,3 glucan synthase and responsible for the biosynthesis of a potent immunological activator, i.e., β-glucan (Ujita et al. 2006). Another group isolated Cu, Zn SOD 1 gene (SOD 1) from *Cordyceps militaris* which not only acts as an anti-oxidant and anti-inflammatory agent but also neutralizes free radicals which could be a potential anti-aging drug (Park et al. 2005). From *Cordyceps sinensis*, two cuticle degrading serine protease genes, i.e., *csp 1* and *csp 2* have been cloned and expressed in yeast *Pichia pastoris.* The genes, *csp1* and *csp 2* were further characterized using synthetic substrate N-suc-AAPF-p-NA to understand the pathobiology and infection to the host (Zhang et al. 2008). Similar studies were carried out to clone and analyse glyceraldehyde-3-phosphate-dehydrogenase (GPD) gene from *Cordyceps militaris*. GPD is an important enzyme used in the glycolytic pathway, which catalyses the phosphorylation of glyceraldehyde-3-phosphate to form 1, 3-diphosphoglycerate, an important reaction to maintain life activities in a cell for the generation of ATP (Gong et al. 2009). Further studies could be directed toward improving *Cordyceps* sp. by developing an effective transformation system.

## Pharmaceutical and therapeutic potential of *Cordyceps* sp.

*Cordyceps* species is also known as traditional Chinese medicine (TCM) as it has wide applications in pharmaceutical (Table 2) and health sector (Ng and Wang 2005; Russell and Paterson 2008). This medicinal mushroom was in the limelight during the Chinese National Games in 1993, when a group of women athletes broke nine world records, committed that they had been taking *Cordyceps* regularly. It has been seen previously reported that *Cordyceps* also enhances physical stamina making it very useful for the elderly people and athletes. Recent literature further confirms that *Cordyceps* enhances cellular energy in the form of ATP (adenosine tri-phosphate). Upon hydrolysis of phosphates from ATP, lots of energy is released which is further used by the cell (Dai et al. 2001; Siu et al. 2004). The studies by many researchers in the past on *Cordyceps* have demonstrated that it has anti-bacterial, anti-fungal, larvicidal, anti-inflammatory, anti-diabetic, anti-oxidant, anti-tumor, pro-sexual, apoptotic, immunomodulatory, anti-HIV and many more activities (Table 2).

**Table 2:** Summary of various pharmacological and therapeutic effects of *Cordyceps* sp.

Pharmacological effect	Active content of *Cordyceps*	Animal/tissue studied	Active dose	Experimental time period	References
Anti-angiogenic	*Cordyceps militaris* Extract (CME)	HUVECs	100–200 mg/L	After 3–6 h	Yoo et al. (2004)
Anti-tumor/anti-proliferatory	*Cordyceps militaris* protein (CMP)	MCF-7 (breast cancer), 5637 (bladder cancer) and A-549 (lung cancer)	15 μM	72 h	Park et al. (2009a, b, c)
	Aqueous extract of *C. militaris*	Nude mice with NCI-H460 cell	At 150 and 300 mg/kg/day	4 weeks	Park et al. (2009a, b, c)
	BuOH extracts of *C. militaris* grown on germinated soybean (GSC)	HT-29 human colon cancer	100 μg/ml	48 h	Mollah et al. 2012
	Cordycepin	Mice	150 mg/kg body weight	7 days	Jagger et al. (1961)
		5637 and T-24 (bladder cancer) KB and HSC3 (oral squamous cell carcinoma)	200 μm	24 h	Lee et al. (2011a, b)
			50 and 30 μM, respectively	48 h	
Anti metastasis	WE of *C. sinensis*	LLC and B16 cells	100 mg/kg in LLC, 100 or 200 mg/kg in B16	20 and 26 days	Nakamura et al. (1999)
	Cordycepin	5637 and T-24 cells	100 and 200 μM	48 h	Lee et al. 2010
Induce apoptosis	etOAc extract of *C. sinensis*	HL-60 cells	ED50 ≤25 μg/ml	2 days	Zhang et al. (2004)
	Aqueous extract of *C. militaris*	MDA-MB-231	0.8 mg/ml	24 h	Jin et al. (2008)
	*Paecilomyces hepiali* (derivative of *C. sinensis*) extract	A549	2–4 mg/ml	48–72 h	Thakur et al. (2011)
	Water extract of *C. militaris*	A549	2 μg/ml	48 h	Park et al. (2009a, b, c)
	Cordycepin	MA-10	100 μM to 5 mM	24 h	Jen et al. (2008)
		SW480 & SW620	2 and 0.72 mmol/L, respectively	72 h	He et al. (2010)
		MDA-MB-231	100 μM	24 h	Choi et al. (2011)
		U937 and THP-1	30 μg/ml	24 h	Jeong et al. (2011)
		SK-NBE(2)-C and SK-Mel-2 (HTB-68)	120 and 80 μM, respectively	24 h	Baik et al. (2012)
Anti fatigue	Polysaccharide	Mice	200 mg/kg	For 21 days	Li and Li (2009)
Anti malaria	Cordycepin	Erythrocytic stages of *P. knowlesi* (in vitro) and *P. berghei* (in vivo)	In vitro 106 M and in vivo 50 mg/kg	In vitro 4 h	Trigg et al. (1971)
Anti fungal	Cordycepin	Murine Model	1.5 mg/kg/day	30 days	Sugar and Mccaffrey (1998)
Hypolipidemic	Exo polysaccharide	Rats	50–100 mg/kg	2 weeks	Yang et al. (2000)
Increase hepatic energy metabolism and blood flow	*Cordyceps sinensis* Extract	Mice	200 mg/kg/daily	4 weeks	Manabe et al. (2000)
Immunomodulatory	Polysaccharide from *C. sinensis*	Human peripheral blood	0.025–0.1 mg	–	Kuo et al. (2007)
	Purified Cordycepin from *C. militaris*	Mouse splenocytes	5 μg/ml	72 h	Ho et al. (2012)
Anti inflammatory	*C. militaris* water extract	Murine macrophage	1,250 μg/ml	24 h	Jo et al. (2010)
	Constituents isolated from *C. militaris*	LPS/IFN-γ stimulated Macrophage cells	Ranging from 6.3 to 20 μg/ml	24 h	Rao et al. (2010)
Anti Diabetic/Hypoglycemic	*C. militaris* extract reduce oxidative stress, induced by high glucose concentration	HUVECs	25 μg/ml	12–36 h	Chu et al. (2011)
	Fractions of *C. militaris* as CMESS and Cordycepin	Mice	50 and 0.2 mg/kg, respectively	7 days	Yun et al. (2003)
	Crude extract and polysaccharide rich fraction	Rat	10 mg/kg of polysaccharide and 100 mg/kg body weight of crude extract	4 days	Zhang et al. (2006)
Spermatogenic	CM mycelium powder	Sub fertile boars	10 g/boar	2 months	Lin and Tsai (2007)
Steroidogenesis	CS	Normal mouse leydig cells	3 mg/ml	2–3 h	Huang et al. (2001)
	Cordycepin	MA-10 mouse leydig tumor cells	100 μM	24 h	Pan et al. (2011)
Anti-aging	CSE	Mice	2.0, 4.0 g/kg	6 weeks	Ji et al. (2009)
	Cordycepin	Human dermal fibroblasts	50–100 μM	24 h	Lee et al. 2009a, 2009b
Anti-fibrotic	EPC from *C. militaris*	Rats	30 mg/kg/day	4 weeks	Nan et al. (2001)
Cardiovascular effects	Cs-4			1–15 min	Zhu et al. (1998b)
Relax aorta		Isolated aorta	50 μg/ml		
Lower blood pressure		Dogs	60 mg/kg		
Increase coronary blood flow		Dogs	0.425 g/kg		
Lower heart rate		Dogs	0.425 g/kg		
Against arrhythmia		Dogs	0.25–0.5 g/kg		
Against myocardial ischemia		Rabbits	150 mg/kg		
Against platelet aggregation		Platelet	2–4 mg/ml		
Against thrombosis		Rabbits	30 μg/kg/min		
Renal protection	*Cordyceps* Powder	LN Patients	2–4 g/day cordyceps powder, and artemisinin 0.6 g/day	3 years and observed consecutively for 5 years	Lu (2002)
Erythropoiesis	*Cordyceps sinensis* crystal (CS-Cr)	LACA Mouse, in vivo and vitro	>150 mg/kg (vivo) 150–200 μg/ml (vitro)	5 consecutive daily treatment	Li et al. (1993)

*Cordyceps* has a long history of use as a lung and kidney tonic, and for the treatment of chronic bronchitis, asthma, tuberculosis and other diseases of the respiratory system. The cardiovascular effects of *Cordyceps* are being noticed more frequently by researchers as it works through variety of possible ways either by lowering high blood pressure via direct dilatory effects or mediated through M-cholinergic receptors resulting in improvement in the coronary and cerebral blood circulation (Zhu et al. 1998b). Thus, *Cordyceps* has implications at the therapeutic level as well by rectifying the abnormalities in rhythmic contractions (also known as cardiac arrhythmia). *Cordyceps* extract has also been found as a promising source to increase cardiac output up to 60 % in augmentation with conventional treatment of chronic heart failure (Chen 1995). The product from wild type and cultured *Cordyceps* has also been shown to significantly decrease blood viscosity and fibrinogen levels preventing myocardial infarction (Zhu et al. 1998b). Another study showed that the fermentation products of Cs-4 reduce myocardial oxygen consumption in animals under experimental lab conditions revealing dramatic anti-anoxic effects (Zhu et al. 1998a). These studies provide strong evidence that Cs-4 and its fermentative solution prevent platelet aggregation stimulated by collagen or adenosine di-phosphate (ADP). An intravenous injection of concentrated *Cordyceps* extract (90 μg/kg per min, i.v.) resulted in 51–71 % reduction in ^51^Cr-labeled platelet aggregation in the endothelial abdominal aorta in rabbit (Zhu et al. 1998b).

## Toxicological and dosage related studies of *Cordyceps*

*Cordyceps* is one of the best medicinal fungi known for numerous positive aspects in terms of pharmacological effects and considered to be safe. Some reports are published on its adverse gastrointestinal behaviors like dry mouth, nausea and diarrhea (Zhou et al. 1998). In some patients, allergic response has been seen during treatment with a strain of *Cordyceps*, i.e., CS-4 (Xu 1994). Patients, who suffer from autoimmune diseases such as rheumatoid arthritis, systemic lupus erythematosus and multiple sclerosis, are generally suggested to avoid its use. Reports are still lacking on pregnant and lactating women but some animal studies in mice have revealed that *Cordyceps* have effects on plasma testosterone levels (Huang et al. 2004; Wong et al. 2007). There has been couple of reports on lead poisoning in patients taking *Cordyceps* herbal medicine for treatment. The lead content in the C*ordyceps* powder in these cases was significantly high (20,000 ppm) (Wu et al. 1996). However, the blood lead levels returned to normal upon termination of the product consumption.

Besides few negatively published data, *Cordyceps* is relatively considered to be a non-toxic medicinal mushroom. *Cordyceps* dose in patients suffering from long-term renal failure was demonstrated up to 3–6 g/day (Zhu et al. 1998b). In clinical studies involving lung cancer, chemotherapy was carried out with the combination of *Cordyceps* (Holliday and Cleaver 2008). In another clinical trial, results of *Cordyceps* (3.15 g for 5 weeks) were compared with placebo to evaluate its effects on physical performance (Parcell et al. 2004). In general, researchers demonstrated that 3–4.5 g of *Cordyceps*/day is sufficient except in patients suffering from severe liver disease (Mizuno 1999). However, no human toxicity report was found and even animal models were failed to determine median lethal dose. *Cordyceps* dosage up to 80 g/kg body weight/day for 7 days was injected intraperitonealy in mice and even then it did not cause any fatality (Li et al. 2006). In another study, rabbits fed through mouth for 3 months at a dose of 10 g/kg/day did not show any deviancy in blood reports, or in kidney, liver functioning (Huang et al. 1987). Even water extract of *Cordyceps sinensis* was found to be non-toxic on macrophage cells line RAW264.7 proliferation (Mizuha et al. 2007). It is suggested that caution should be taken while taking *Cordyceps* by patients who are undergoing anti-viral or diabetic drug treatments as Cordyceps contains hypoglycemic and anti-viral agents, which can further affect the dosage of these drugs (Holliday and Cleaver 2008).

## Future perspective

**Fig. 5:**
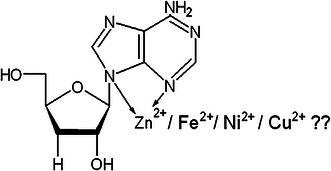
Proposed metal complexes of Cordycepin which could be formed with various transition metals ion

*Cordyceps* is a natural medicinal mushroom which is well liked by people nowadays as they believe more in natural therapy than chemotherapy because of lesser side effects. Growth characteristics of *Cordyceps militaris* have to be studied in-depth to cultivate this mushroom for its mass-scale production so that one could collect enough bio-metabolites from its mycelium extract. There is a strong urge to use interdisciplinary biotechnological and chemical tools to isolate and enhance the bioactivity of the metabolites from this entomopathogenic fungus. The structure of Cordycepin suggests that it has five N and three O atoms which one can imagine could form transition metal complexes in the form of di-, tri- and tetra-dentate ligands as metals can accommodate donor atom’s lone pair of electrons into their empty *d* orbital (Fig. 5). Complexity of the resulting compound and its molecular mass can be predicted with the help of spectroscopic tools like IR and mass spectroscopy, respectively, which can further improve the bioactivity of the compounds.

The remaining pharmacologically active compounds apart from Cordycepin also need to be identified and elucidate their structure–function relationship.

## Conclusions

The usage of natural/herbal medicines over the synthetic ones has seen an upward trend in the recent past. *Cordyceps* being an ancient medicinal mushroom used as a crude drug for the welfare of mankind in old civilization is now a matter of great concern because of its unexplored potentials obtained by various culture techniques and being an excellent source of bioactive metabolites with more than 21 clinically approved benefits on human health including anti-diabetic, anti-tumor, anti-oxidative, immunomodulatory, sexual potentiator and anti-ageing effects (Das et al. 2010b). Cordycepin alone has been widely explored for its anti-cancer/anti-oxidant activities, thus, holding a strong pharmacological and therapeutic potential to cure many dreadful diseases in future. Further investigations need to be focused on to study the mechanistic insight into the mysterious potential of this medicinal mushroom on human health and promoting its cultivation strategies for commercialization and ethno-pharmacological use of this wonderful herb.
